# 1,25-Dihydroxyvitamin D3 suppresses CD4^+^ T-cell effector functionality by inhibition of glycolysis

**DOI:** 10.1111/imm.13472

**Published:** 2022-03-28

**Authors:** Emma L. Bishop, Nancy H. Gudgeon, Gillian M. Mackie, Daniel Chauss, Jennie Roberts, Daniel A. Tennant, Kendle M. Maslowski, Behdad Afzali, Martin Hewison, Sarah Dimeloe

**Affiliations:** 1 Institute of Immunology and Immunotherapy, University of Birmingham, Birmingham, UK; 2 Immunoregulation Section, Kidney Diseases Branch, National Institute of Diabetes and Digestive and Kidney Diseases (NIDDK), National Institutes of Health, Bethesda, Maryland, USA; 3 Institute of Metabolism and Systems Research, University of Birmingham, Birmingham, UK

**Keywords:** immunometabolism, glycolysis, metabolism, T cell, vitamin D

## Abstract

In CD4^+^ T helper cells, the active form of vitamin D_3_, 1,25-dihydroxyvitamin D_3_ (1,25D) suppresses production of inflammatory cytokines, including interferon-gamma (IFN-γ), but the mechanisms for this are not yet fully defined. In innate immune cells, response to 1,25D has been linked to metabolic reprogramming. It is unclear whether 1,25D has similar effects on CD4^+^ T cells, although it is known that antigen stimulation of these cells promotes an anabolic metabolic phenotype, characterized by high rates of aerobic glycolysis to support clonal expansion and effector cytokine expression. Here, we performed in-depth analysis of metabolic capacity and pathway usage, employing extracellular flux and stable isotope-based tracing approaches, in CD4^+^ T cells treated with 1,25D. We report that 1,25D significantly decreases rates of aerobic glycolysis in activated CD4^+^ T cells, whilst exerting a lesser effect on mitochondrial glucose oxidation. This is associated with transcriptional repression of Myc, but not repression of mTOR activity under these conditions. Consistent with the modest effect of 1,25D on mitochondrial activity, it also did not impact CD4^+^ T-cell mitochondrial mass or membrane potential. Finally, we demonstrate that inhibition of aerobic glycolysis by 1,25D substantially contributes to its immune-regulatory capacity in CD4^+^ T cells, since the suppression of IFN-γ expression was significantly blunted in the absence of aerobic glycolysis. 1,25-Dihydroxyvitamin D_3_ (1,25D) suppresses the production of inflammatory cytokines such as interferon-gamma (IFN-γ) by CD4^+^ T cells, but the underpinning mechanisms are not yet fully defined. Here, we identify that 1,25D inhibits aerobic glycolysis in activated CD4^+^ T cells, associated with decreased c-Myc expression. This mechanism appears to substantially contribute to the suppression of IFN-γ by 1,25D, since this is significantly blunted in the absence of aerobic glycolysis.

## INTRODUCTION

The active form of vitamin D_3_, 1,25-dihydroxyvitamin D_3_ (1,25D), has modulatory activity on a range of immune cells, prompting an interest in its potential therapeutic use in autoimmune and inflammatory disease [1,2]. Although a systemic steroid hormone, 1,25D is also synthesized by antigen-presenting cells (APCs), which also express the nuclear vitamin D receptor (VDR) for 1,25D [1]. Within an immune microenvironment, 1,25D can therefore act in an intracrine fashion to modulate APC activity by suppressing expression of co-stimulatory molecules, such as CD80/86, whilst increasing expression of inhibitory molecules such as PD-L1 [3,4]. Induction of a tolerogenic APC phenotype in this way, enables 1,25D to indirectly suppress T cell function and promote regulatory T cell (Treg) differentiation [3,5,6]. However, 1,25D can also bind to VDR in T cells to exert direct effects, notably, induction of Treg, upregulation of CTLA-4 and IL-10, and downregulation of inflammatory cytokines such as IL-17 and interferon-gamma (IFN-γ) [2,7–10]. The mechanisms for this direct regulation of T cells by 1,25D are not yet fully characterized.

In recent years, studies have shown that regulation of metabolic function is a key target for 1,25D in innate immune cells [4,5,11,12]. In macrophages, 1,25D can alter cholesterol metabolism to reduce the formation of foam-cells [13,14]. Tolerogenic dendritic cells (TolDCs) differentiated in the presence of 1,25D exhibit high levels of glycolysis and elevated fatty acid synthesis, which enable these DCs to exert their tolerogenic effects on T cells [4,5,11,12]. Cellular metabolism is also closely associated with T cell function. Upon antigen recognition, T cells substantially increase metabolic activity, and particularly their rates of aerobic glycolysis. This supports the clonal expansion of T cells through the rapid generation of ATP, as well as nucleotide, amino acid, and lipid precursors produced from glycolytic intermediates [15,16]. Moreover, increased glycolytic metabolism has been directly linked to the expression of effector cytokines such as IFN-γ, through both transcriptional and post-transcriptional mechanisms [17,18]. Specifically, increased glycolytic activity of the enzyme glyceraldehyde 3-phosphate dehydrogenase (GAPDH) in activated T cells decreases its binding to IFN-γ mRNA, thereby permitting enhanced translation [17]. Additionally, glucose-derived acetyl-CoA, generated via activity of the tri-carboxylic acid (TCA) cycle is used to acetylate histones, facilitating IFNG gene transcription [18]. Recent studies have suggested a role for 1,25D as a modulator of T cell metabolism, by identifying transcriptional regulation of key metabolic genes [19–21], whilst others have correlated high serum 25D levels with lower rates of mitochondrial oxidative phosphorylation (OXPHOS) and glycolysis in T cells [22,23]. However, specific metabolic effects of 1,25D on T cells and how these relate to immunomodulatory activity have yet to be studied in detail. We therefore set out to interrogate the effects of 1,25D on human CD4^+^ T cell metabolism, through in-depth analysis of functional metabolic capacity and metabolic pathway usage.

## MATERIALS AND METHODS

### Human peripheral blood donors

Human CD4^+^ T cells were isolated from fully anonymized leukocyte cones collected from NHS Blood and Transplant (NHSBT), Birmingham, UK. For qPCR analysis, cells were isolated from the peripheral blood of anonymous healthy volunteers. All volunteers signed a consent form, and all studies were approved by the University of Birmingham STEM Ethics Committee (Ref. ERN 17_1743).

### Peripheral blood CD4^+^ T cell isolation and culture

Human primary CD4^+^ T cells were isolated as described previously [24]. Purity was typically >95%. Cells were (unless otherwise indicated) resuspended at 1 × 10^6^/ml in RPMI-1640 (Gibco, Cat# 21875034) containing 10% fetal calf serum (Sigma-Aldrich, Cat# F9665), 50 U/ml penicillin and 50 mg/ml streptomycin (Thermo Fisher Scientific, Cat# 15140122) (RPMI/10% FCS), and 50 IU/ml rIL-2 (unless otherwise indicated) (PeproTech, Cat# 20002). In experiments requiring glucose-free media, CD4^+^ T cells were cultured in RPMI-1640 without glucose (Gibco, Cat# 11879020) containing 10% FCS, 50 U/ml penicillin and 50 mg/ml streptomycin, and rIL-2 (50 IU/ml). Where indicated in the figure legends, D(+)-Glucose (10 mM; Sigma-Aldrich, Cat# G7021), D(+) Galactose (10 mM; Sigma-Aldrich, Cat# G5388), sodium pyruvate (10 mM; Alfa Aesar, Cat# J61840), or sodium acetate (5 mM; Sigma-Aldrich, Cat# S5636), isotype control or anti-IL-2 antibodies (both 5 μg/ml; BioLegend, Cat# 400543/500353) were added to culture. Cells were activated using ImmunoCult Human CD3/CD28 T-Cell Activator (12 μl/ml; STEMCell Technologies, Cat# 10991) for the time indicated in figure legends. All treatments were added at the beginning and remained present for the duration of culture, 1,25-dihydroxyvitamin D_3_ (1–10 nM; Enzo Life Sciences, Cat# BML-DM200) and rapamycin (20 ng/ml; Merck, Cat# 553210).

### Flow cytometric analysis of metabolism, protein expression, and phosphorylation

After culture, CD4^+^ T cells (0.2 × 10^6^) were stained with metabolic probes diluted in T-cell culture media for 20 min at 37° celsius. Cells were then washed twice in staining buffer (PBS/2% FCS) and resuspended before analysis. Metabolic probes used included MitoSpy Orange (MSO) (25 nM; BioLegend, Cat# 424803), and MitoView Green (MVG) (50 nM; Biotium, Cat# 70054). For analysis of c-Myc expression and p70S6K phosphorylation, cells (0.2 × 10^6^) were first fixed in FoxP3 fixation/permeabilization solution (eBioscience, Cat# 005523–00) for 20 min at 37° celsius, washed in FoxP3 permeabilization buffer and incubated with anti-c-Myc (Cell Signalling Technology, Cat# 5605T) or anti-phospho p70S6K (Thr421/ Ser424) (Cell Signalling Technology, Cat# 9204S) for 30 min at 4° celsius. After washing, cells were then incubated with secondary antibody anti-Rabbit IgG (H + L), AF555 (Invitrogen, Cat# A-21428) for 20 min at 4° celsius, followed by a final wash. Samples were run on the BD LSRFortessa X-20, data collected using BD FACSDiva Software, and analysed using FlowJo version 10.7.1 (BD Biosciences).

### Measuring cytokine production

For measurement of cytokine production by CD4^+^ T cells in culture, supernatants were collected at 48 hr or 5 days and stored at −20° celsius until analysis. Cytokines were measured using LEGENDplex Human Th Cytokine Panel (12-plex) (BioLegend, Cat# 741028), and IFN-γ levels measured by enzyme-linked immunosorbent assay (ELISA) using anti-IFN-γ capture (Bio-Rad, Cat# HCA043) and biotinylated detection (Bio-Rad, Cat# HCA044P) anti-bodies, recombinant IFN-γ standard (Bio-Rad, Cat# PHP050), streptavidin-HRP (Sigma-Aldrich, Cat# E2866), and TMB substrate (Bio-Rad, Cat# BUF062B).

### Extracellular metabolic flux analysis

For analysis of the oxygen consumption rates (OCR) and extracellular acidification rates (ECAR), the Seahorse XFe96 metabolic extracellular flux analyser was used (Agilent Technologies). CD4^+^ T cells were resuspended in Seahorse XF RPMI medium (Agilent Technologies, Cat# 103576100) containing D-(+)-Glucose (10 mM), sodium pyruvate (1 mM), and L-Glutamine (5 mM; Sigma-Aldrich, Cat# G8540), and were plated onto Sea-horse cell plates (2.5 × 10^5^ cells per well) coated with Cell-Tak (BD Biosciences) to enhance T-cell attachment. Perturbation profiling of the use of metabolic pathways was done by the addition of Oligomycin (1 μM), BAM 15 (3 μM) and Rotenone/Antimycin A (2 μM/2 μM); all are given as final concentrations, all from Sigma-Aldrich). Using this perturbation profiling technique, four OCR rates were directly measured: the basal OCR [OCR(Basal)], the rate after inhibition of ATP-synthase with Oligomycin [OCR(Oligomycin)], the peak rate after mitochondrial uncoupling with BAM 15 [OCR(BAM 15)], and the rate after inhibition of mitochondrial respiration with Rotenone/Antimycin A [OCR(Rotenone/Antimycin A)]. The following respiratory parameters were then calculated from using the following formulae: (i) Basal OCR = [OCR (Basal)] – [OCR(Rotenone/Antimycin A)]; (ii) ATP-coupled respiration (OCR) = [OCR(Basal)] – [OCR(Oligomycin)]; (iii) maximal respiratory capacity = [OCR(BAM 15)] – [OCR(Rotenone/Antimycin A)]; and (iv) spare respiratory capacity (SRC) = [OCR(BAM 15)] – [OCR(Basal)]. Basal ECAR was determined as the initial rate as measured by the extracellular flux analyser and the maximal ECAR was the rate after the addition of Oligomycin.

### Stable isotope glucose tracing

From cell culture, cells were resuspended in SILAC RPMI-1640 Flex Media (Gibco, Cat# A2494201) containing 10% FCS, rIL-2 (50 IU/ml), L-Lysine (4 μg/ml; Sigma-Aldrich, Cat# L5501), L-Arginine (20 μg/ml; Sigma-Aldrich, Cat# A8094), and L-Glutamine (200 mM). U-13C-labelled D-glucose (10 mM; Cambridge Isotopes) was added to each well and the cells incubated for 4 hr at 37° celsius. A number of 4 × 10^6^ cells per condition were labelled as indicated, then washed with ice-cold 0.9% saline solution and were extracted in 1:1:1 pre- chilled methanol, HPLC-grade water (containing 1 μg/ml D6-glutaric acid) and chloroform. The extracts were shaken at 400 *g* for 20 min at 4° celsius and centrifuged at 16 000*g* for 5 min at 4° celsius. The upper aqueous phase (0.3 ml) was collected and evaporated under vacuum. Metabolite derivatization was performed using an Agilent autosampler. Dried polar metabolites were dissolved in 15 μl of 2% methoxyamine hydrochloride in pyridine (Thermo Fisher Scientific, Cat# 25104) at 55° celsius, followed by an equal volume of N-tert-Butyldimethylsilyl-N-methyltrifluoroacetamide with 1% tertbutyldimethylchlorosilane after 60 min, and incubation for a further 90 min at 55° celsius. Gas chromatography–mass spectrometry (GC–MS) analysis was performed using an Agilent 6890GC equipped with a 30-m DB-35MS capillary column. The GC was connected to an Agilent 5975C MS operating under electron impact ionization at 70 eV. The MS source was held at 230° celsius and the quadrupole at 150° celsius. The detector was operated in scan mode and 1 μl of derivatized sample was injected in splitless mode. Helium was used as a carrier gas at a flow rate of 1 ml/min. The GC oven temperature was held at 80° celsius for 6 min and increased to 325° celsius at a rate of 10° celsius/min for 4 min. The run time for each sample was 59 min. For determination of the mass isotopomer distributions (MIDs), spectra were corrected for natural isotope abundance. Data processing was performed using MATLAB.

### Quantification of mRNA

Selected genes of interest were quantified using real-time reverse transcriptase quantitative polymerase chain reaction (qPCR). From cell culture, CD4^+^ T cells were harvested, and mRNA isolated using NucleoSpin RNA Mini Kit (MACHEREY-NAGEL, Cat# 740955.5) according to the instructions. The cDNA was transcribed using Promega M-MLV Reverse Transcriptase (Cat# M1701), RNasin Plus Ribonuclease Inhibitor (Cat# N2611), oligo(dT) 15 Primer (Cat# C1101), and PCR Nucleotide Mix (Cat# C1141). SYBR Green PCR Master Mix (Applied Biosystems, Cat# 4309155) was used for qPCR analysis, the primers used are detailed in Table S1.

### Western blot analysis of proteins

After 48 hr of cell culture, CD4^+^ T cells were harvested and lysed using RIPA buffer with protease and phosphatase inhibitors. BCA Protein Assay Kit (Thermo Fisher Scientific, Cat# 23225) was used to determine protein concentration according to the manufacturer’s instructions and samples adjusted to contain 30 μg of protein in Laemmeli Sample Buffer (Sigma-Aldrich, Cat# S3401) containing β mercaptoethanol (Sigma-Aldrich, Cat# 63689). Cell lysates were separated by 10% SDS-polyacrylamide gel electrophoresis and transferred onto nitrocellulose mem- branes using iBlot Transfer Stack (Invitrogen, Cat# IB301002) and Thermo Fisher Scientific iBlot Gel Transfer Device. The membranes were then incubated with primary anti phospho-p70S6K, followed by secondary antibody goat anti-Rabbit. Bands were detected using Clarity Western ECL Substrate (Bio Rad, Cat# 1705061) and imaged using the Bio Rad ChemiDoc Imaging System.

### Confocal analysis of mitochondria

Fixed cells were viewed and imaged with a Zeiss LSM780 confocal microscope and Plan-ACHROMAT ×40 objective lens (digital zoom used in closer images). CD4^+^ T cells were allowed to attach by gravity for 20 min onto culture slides (BD Bioscience) coated with poly-D-lysine, during which time they were also stained with MitoSpy Orange as described above. Attached cells were then washed twice, fixed with 4% paraformaldehyde and permeabilized with 0.3% Triton X-100. Cells were stained with DAPI (4,6-diamidino-2-phenylindol) for nuclear staining. Stacked images were then collected at intervals of 0.5 or 1 μm. All images were processed and analysed with ZEN 3.1 blue edition software.

### Statistical analysis

All data were analysed using GraphPad Prism 9 (GraphPad Software). Data are presented as mean standard error of the mean (SEM) and significance tested using Student’s t-test. For multiple groups, analysis was performed using one-way analysis of variance (ANOVA) with Dunnett’s post-hoc test. For data normalized to the control, the median is presented and non-parametric Kruskal–Wallis with Dunn’s post-hoc test for multiple comparisons was used for statistical analysis.

## RESULTS

### 1,25D inhibits the metabolic activity of activated T cells

**FIGURE 1:**
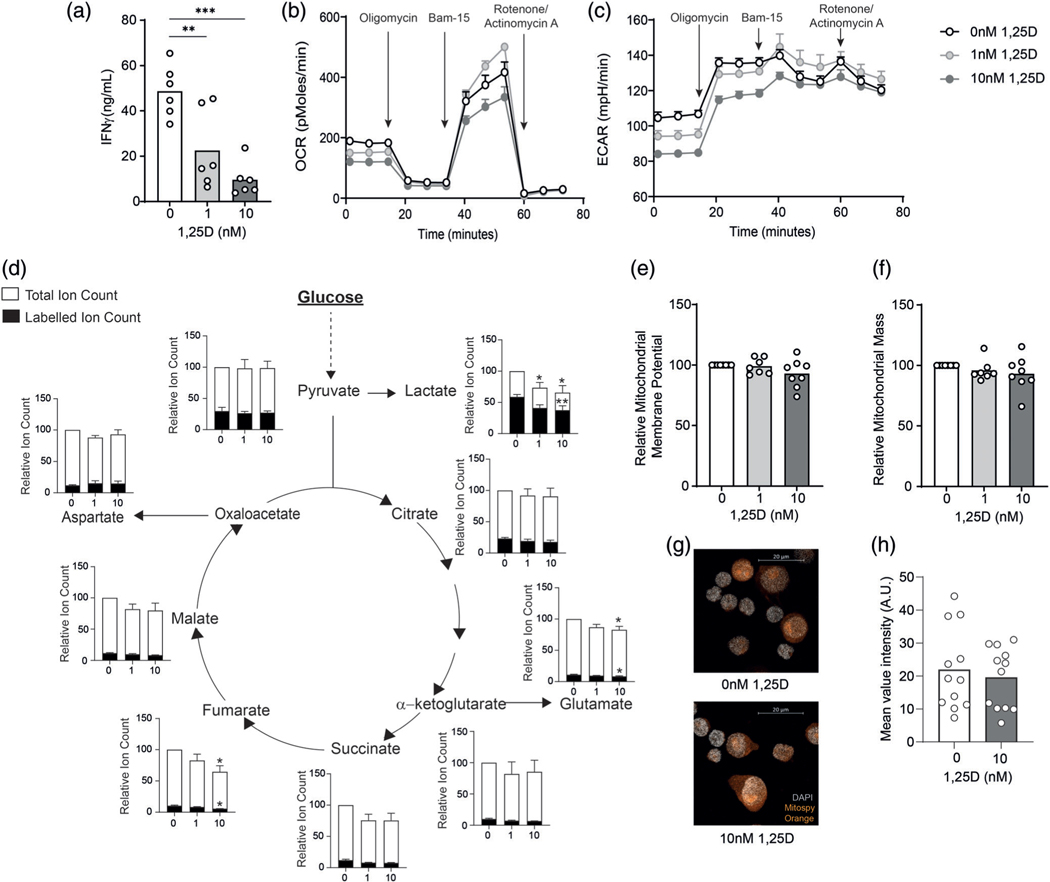
1,25D inhibits metabolic activity of CD4^+^ T cells. (a) Human primary CD4^+^ T cells were stimulated in presence of 0, 1, or 10 nM 1,25D as indicated for 48 hr and assessed for IFN-γ secretion by ELISA analysis of cell culture supernatants (*n* = 6 independent donors). (b,c) Human primary CD4^+^ T cells, cultured as in (a) were assessed for oxygen consumption rates (a, OCR) and extracellular acidification rates (b, ECAR) by extracellular flux analysis. Arrows indicate injection time points of the indicated compounds (see Section 2 for details) (one representative example from three independent donors, error bars indicate technical replicates). (D) CD4^+^ T cells, activated as in (a) were washed and then incubated with fully 13C-labelled glucose for 6 hr and assessed for total and 13-C-labelled ion counts of the indicated metabolites by GC–MS (*n* = 5 independent donors, data for 1,25D-treated cells are normalized to non-treated controls within each experiment). (e,f) Human primary CD4^+^ T cells, cultured as in (a) were assessed for mitochondrial membrane potential (e) and mitochondrial mass (f) by flow cytometry analysis of MitoSpy Orange and MitoView Green staining respectively (*n* = 7 independent donors, data for 1,25D-treated cells are normalized to non-treated controls within each experiment). (g) Mitochondria were visualized by MitoSpy Orange staining and confocal microscopy (h) Mean intensity of MitoSpy Orange staining per cell within confocal images (*n* = 2 independent donors) *p < 0.05, **p < 0.01, ***p < 0.001

We activated human CD4^+^ T cells in the presence of 0, 1, or 10 nM 1,25D for 48 hr and measured IFN-γ levels by ELISA to confirm functional suppression by 1,25D in our culture system. 1,25D suppressed levels of IFN-γ in CD4^+^ T cells in a dose-dependent manner after 48 hr of culture (Figure 1a). This effect was maintained by Day 5 of culture with a trend towards increased IL-10 expression (Figure S1A). No differences in Th2 cytokines were seen with 1,25D treatment (Figure S1B).

As IFN-γ expression is known to be controlled by metabolic activity of T cells [17,18], we next investigated whether the effects of 1,25D on CD4^+^ T cell IFN-γ expression were associated with changes in metabolic function. Activated CD4^+^ T cells cultured in the presence of 0, 1, or 10 nM 1,25D for 48 hr were assessed for respiratory and glycolytic capacity by extracellular flux analysis. Both 1 and 10 nM 1,25D decreased the basal and ATP-coupled OCR of these cells but had less effect on maximal and spare respiratory capacity, stimulated by injection of the mitochondrial uncoupling reagent, Bam-15 (Figure 1b). CD4^+^ T cells treated with 1 or 10 nM 1,25D also demonstrated decreased basal ECAR, indicating reduced lactate production from glucose, as well as decreased maximal ECAR rates following oligomycin injection (Figure 1c).

To interrogate glucose metabolism in more detail, we employed a stable isotope-based tracing approach. CD4^+^ T cells that had been activated in presence or absence of 1,25D were incubated with fully-labelled 13C-glucose before analysis of metabolite abundances and 13C-labelling by GC–MS. These experiments identified no significant difference in the fractional 13C-labelling of pyruvate, or of overall pyruvate abundance with either dose of 1,25D (Figure 1d). However, both total and labelled lactate were significantly decreased upon treatment with 1,25D. In all TCA cycle intermediates there was a trend towards a reduction in both total and labelled ion counts with 1,25D treatment, but this was only significant for fumarate and glutamate, which is synthesized from α-ketoglutarate (Figure 1d). These data indicate that 1,25D significantly suppresses aerobic glycolysis and lactate production in T cells, and, to a lesser extent, mitochondrial glucose oxidation. In agreement with this finding, no significant difference in mitochondrial membrane potential was observed in 1,25D-treated T cells, as measured using the membrane-potential sensitive fluorescent probe MitoSpy Orange (Figure 1e). Likewise, use of MitoView Green to measure mitochondrial mass by flow cytometry also showed no difference with 1,25D treatment (Figure 1f). To confirm this by another approach, mitochondria were visualized by confocal microscopy, which also confirmed no change in overall cellular mitochondrial content (Figure 1g,h). Glucose tracing data also indicated little incorporation of glucose into serine in the control or 1,25D-treated T cells under these conditions (Figure S2A), as well as no change in the transcription of rate-limiting enzyme phosphoglycerate dehydrogenase, suggesting no divergence to one-carbon metabolism in CD4^+^ T cells treated with 1,25D (Figure S2B).

### 1,25D suppresses c-Myc expression in activated T cells, but not mitochondrial biogenesis

**FIGURE 2:**
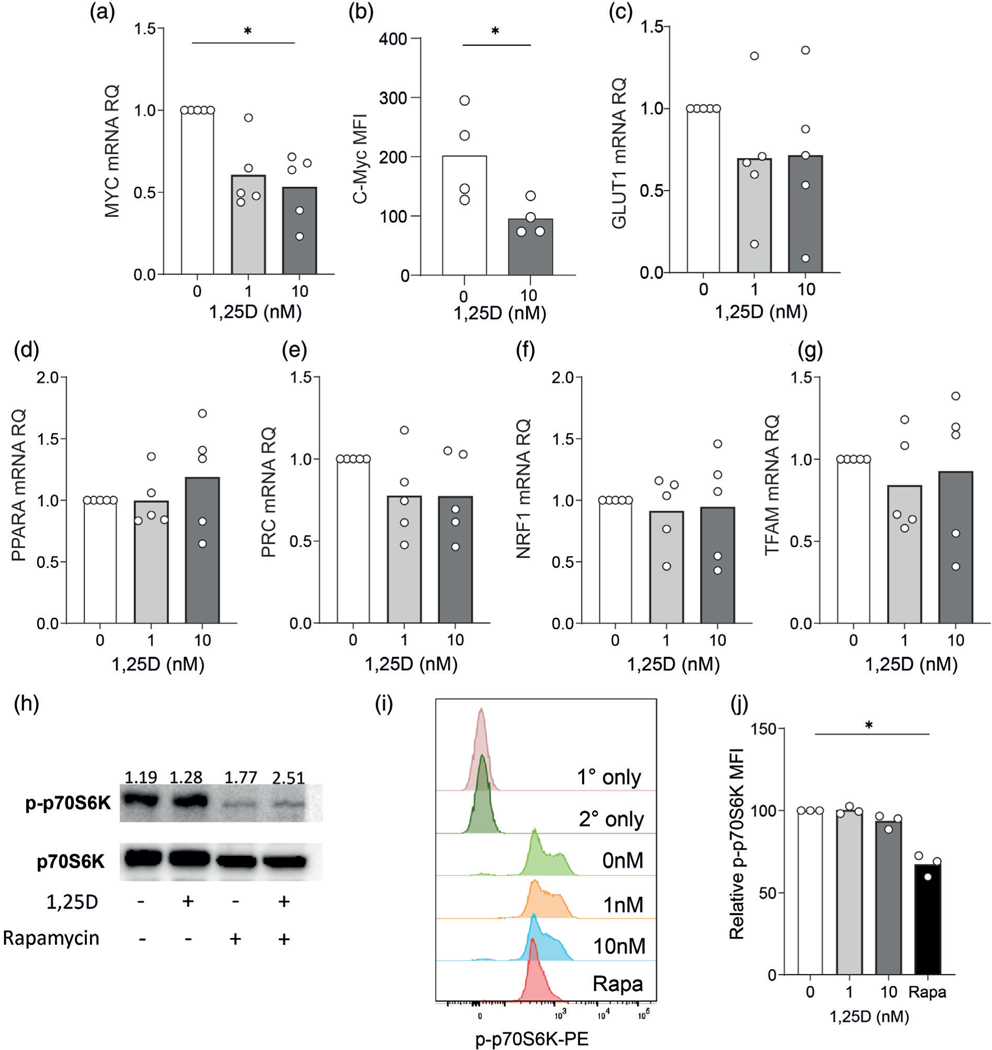
1,25D inhibits expression of c-Myc, but not mitochondrial biogenesis related genes or mTOR activity. (a,b) Human primary CD4^+^ T cells were stimulated in presence of 0, 1, or 10 nM 1,25D as indicated for 48 hr and assessed for (a) Myc mRNA abundance by qPCR and (b) C-Myc protein expression by flow cytometry, expressed as mean fluorescence intensity (MFI). (c–g) Human primary CD4^+^ T cells were stimulated in presence of 0, 1, or 10 nM 1,25D as indicated for 48 hr and assessed for mRNA abundance of the indicated genes by qPCR. (h) CD4^+^ T cells, activated as in (a) with 10 nM 1,25D or 20 ng/ml Rapamycin (Rapa) were analysed for abundance of phosphorylated p70S6K (p-p70S6K) or total p70S6K by western blot (one representative example from three independent donors). (i,j) CD4^+^ T cells, activated as in (a) with 1 or 10 nM 1,25D or 20 ng/ml Rapamycin (Rapa) were analysed for abundance of phosphorylated p70S6K (p-p70S6K) by flow cytometry; (i) shows representative flow cytometry histograms for p-p70S6K under the different conditions and also for the primary and secondary antibody-only controls; (i) data are summarized (*n* = 3 independent donors, data for 1,25D-treated cells are normalized to non-treated controls within each experiment). *p < 0.05

C-Myc is an important transcription factor driving glycolysis and glutaminolysis in T cells [25,26] and expression of c-Myc has been reported to be suppressed by 1,25D in T cells [20,21]. After 48 hr, CD4^+^ T cells treated with 1 or 10 nM 1,25D demonstrated lower levels of Myc mRNA than control cells (Figure 2a). In agreement with this, c-Myc protein levels, as assessed by flow cytometry were also decreased by 50% on average in 1,25D-treated T cells (Figure 2b). One possible explanation for this is that c-Myc expression is decreased secondary to 1,25D-mediated inhibition of IL-2 secretion, since IL-2 is reported to drive c-Myc expression [25]. However, we observed that 1,25D suppressed c-Myc protein expression across a broad range of IL-2 concentration in culture, and to an equal extent in presence of an IL-2 neutralizing antibody as an isotype control (Figure S3A,B). Taken together, these data indicate that 1,25D may directly inhibit c-Myc expression independently of its effects on IL-2. C-Myc is known to induce transcription of the glucose transporter Glut1 [25,26]. Here, analysis of Glut1 mRNA levels did not identify a consistent downregulation upon 1,25D treatment, although a downward trend was observed (Figure 2c). Analysis of a panel of key mitochondrial biogenesis genes (Ppara, Prc, Nrf1, or Tfam) showed no effect of 1,25D on mRNA expression (Figure 2d–g), consistent with the lack of effect of 1,25D on mitochondrial mass and function at this time point. Transcription of Myc in CD4^+^ T cells is induced by the PI3K/Akt/mTOR pathway [25,26]. We therefore sought to assess whether 1,25D impacted mTOR activity. Western blot analysis of phosphorylated p70S6K, a protein kinase directly phosphorylated by mTOR complex 1 (mTORC1), showed no difference with 1,25D treatment (Figure 2h). This was confirmed by flow cytometry, where significant loss of phospho-p70S6K was only observed for cells treated with the mTOR inhibitor rapamycin (Figure 2i,j). These data indicate that the suppressive effect of 1,25D on glycolysis is associated with inhibition of c-Myc, but does not involve inhibition of mTOR activity under these conditions.

### Inhibition of aerobic glycolysis contributes to the immune-suppressive effects of 1,25D

**FIGURE 3:**
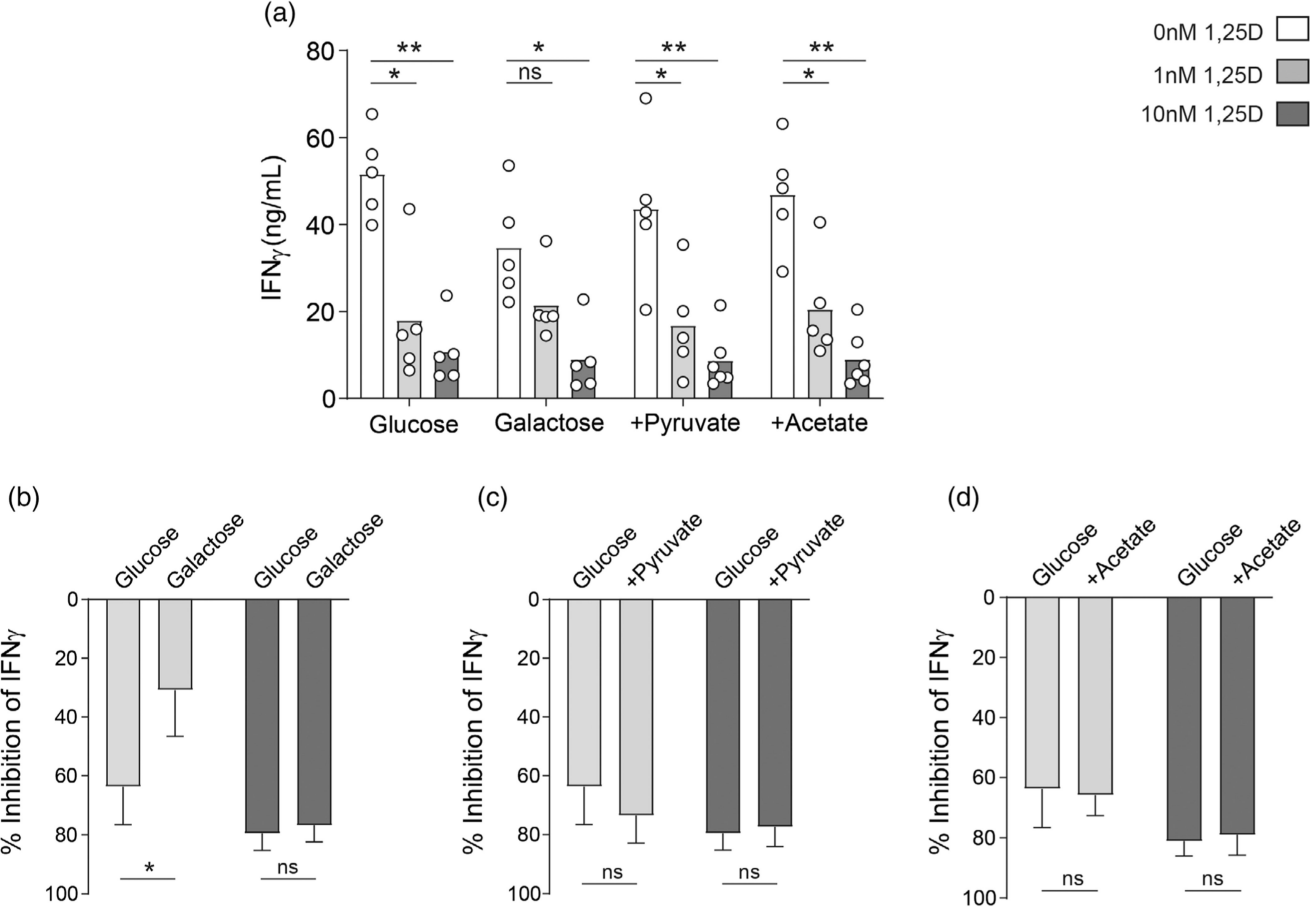
Suppression of aerobic glycolysis contributes to 1,25D-mediated inhibition of IFN-γ expression. (a) Human primary CD4^+^ T cells were stimulated in presence of 0, 1, or 10 nM 1,25D as indicated for 48 hr either in glucose or galactose-containing as indicated (10 mM of either), or in glucose-containing medium additionally supplemented with 10 mM Pyruvate or Acetate as indicated and assessed for IFN-γ secretion by ELISA analysis of cell culture supernatants (*n* = 5 independent donors). (b–d) The percentage inhibition of IFN-γ expression was calculated in comparison to control, untreated cells (0 nM 1,25D) for each cell culture condition. *p < 0.05, **p < 0.01, ***p < 0.001

To determine whether the suppression of glycolysis by 1,25D contributed to its immunomodulatory effects on T cells, CD4^+^ T cells were cultured in the presence of 0, 1, or 10 nM 1,25D in either normal, glucose-containing medium, or medium where glucose had been replaced with 10 mM galactose. Conversion of galactose to glucose consumes two molecules of ATP, so that subsequent glycolysis cannot yield any net ATP. This shifts metabolic activity away from aerobic glycolysis and towards OXPHOS [17]. Studies were therefore carried out to determine if 1,25D is still able to exert suppressive effects on IFN-γ in the absence of aerobic glycolysis. Consistent with previous observations [17], levels of IFN-γ were lower in supernatants of control CD4^+^ T cells cultured in galactose-containing medium than those cultured in normal glucose-containing medium (Figure 3a). Additionally, the inhibitory effect of 1 nM 1,25D on IFN-γ expression was substantially blunted in galactose-containing medium: a mean suppression of 60% in glucose-containing medium was reduced to 25% in galactose-containing medium (Figure 3b). These data indicate that suppression of aerobic glycolysis by 1 nM 1,25D substantially contributes to its inhibition of IFN-γ. This effect was not apparent at 10 nM 1,25D (Figure 3a,b), suggesting that transcriptional repression of the IFNG locus in presence of 1,25D is dominant at this dose. Indeed, at 10 nM 1,25D, we see almost complete suppression of IFN-γ at both an mRNA and protein level, compared to a lesser suppression with 1 nM (Figures 1a and S4A). A recent study has identified several 1,25D-induced transcription factors in T cells that may mediate this transcriptional repression, including c-Jun, STAT3 and BACH2, with BACH2 appearing to play a particularly important role [27]. Consistent with the lack of effect of 1,25D on mitochondrial oxidative capacity (Figure 1b), neither addition of exogenous pyruvate, nor acetate—which can directly substitute for glucose-derived acetyl-CoA to regulate IFN-γ transcription—could rescue the inhibitory effect of 1,25D on IFN-γ expression (Figure 3a,c,d).

## DISCUSSION

It is widely reported that 1,25D can directly inhibit T-cell inflammatory function, including suppression of cytokines such as IFN-γ [8,27,28]. Here, we have shown that this effect is associated with the suppression of glycolytic metabolism by 1,25D in activated CD4^+^ T cells. Employing in-depth metabolic tracing approaches, we found that 1,25D significantly inhibited the conversion of glucose to lactate by aerobic glycolysis in CD4^+^ T cells, and to a lesser extent glucose oxidation in the TCA cycle. This was associated with repression of c-Myc expression, although knockdown studies would be required to confirm a causative role for this. We observed no change in mTOR activity upstream of c-Myc and this suppression also occurred independently of effects of 1,25D on IL-2 expression; however, other regulators of c-Myc, such as T-cell receptor signalling were not investigated. Additionally, the effect of 1,25D may be mediated by direct transcriptional repression of Myc, a mechanism reported in other cell types [29].

Recent studies have identified a positive feedback loop between lactate dehydrogenase A (LDHA) activity and PI3K/Akt signalling in T cells, with LDHA-generated ATP critically supporting PI3K signalling and thereby further promoting metabolic reprogramming [30,31]. Since LDHA expression is controlled by c-Myc, it is possible this feed-forward loop is also inhibited by 1,25D, and would be of interest to interrogate in future studies. Other genes under transcriptional control by c-Myc include those involved in glutaminolysis. Glutamine uptake, metabolism and oxidation in the TCA cycle play a significant role supporting T-cell activation and immune function. Therefore, diminished c-Myc driven activity of this pathway could represent an additional 1,25D-mediated metabolic inhibition in T cells. Whilst we did not investigate this, we did notably observe decreased rates of mitochondrial oxygen consumption in 1,25D-treated cells, despite no clear reduction in glucose oxidation in the TCA cycle. It is plausible therefore that this observed change in mitochondrial oxygen consumption relates to the suppressed oxidation of other TCA cycle substrates, such as glutamine or fatty acids. By activating CD4^+^ T cells in presence of galactose rather than glucose, which effectively inhibits aerobic glycolysis and promotes oxidative metabolism [17], we identified that the suppression of aerobic glycolysis substantially contributes to the inhibitory activity of 1,25D on IFN-γ expression at 1 nM 1,25D, but not at 10 nM. 1,25D exposure leads to repressed transcription of IFN-γ [29,32], through altered expression and activity of several transcription factors, notably BACH2 [27], which is likely the prevailing mechanism seen at the higher concentration. However, with less transcriptional repression at the lower concentration, 1,25D-mediated suppression of aerobic glycolysis is an additional contributor to the inhibition of IFN-γ. As there was little effect of 1,25D on mitochondrial oxidative capacity, and the addition of acetate to the culture was unable to rescue IFN-γ production, it is inferable that this inhibition is mediated post-translationally by GAPDH rather than via reduced acetyl-CoA availability limiting histone acetylation [17,18].

Prior investigations have shown 1,25D promotes fatty acid synthesis, oxidative phosphorylation, c-Myc transcription, and glycolysis in TolDCs during differentiation from monocytes [4,12], this appears in contradiction to the suppressive effects on CD4^+^ T cell metabolism seen here. The mechanisms for these differing effects remain unclear. However, it is probable that 1,25D has different targets in each cell type. For example, c-Myc is induced by 1,25D in TolDCs through increased mTOR activity [4], which we do not see in CD4^+^ T cells. The divergent effects of 1,25D on DC and T cell metabolism may also reflect the adaptability of vitamin D in the setting of antigen-presentation and T cell function. The predominant mode of action for vitamin D in antigen-presenting cells such as DC is intracrine through endogenous generation of 1,25D from precursor 25-hydroxyvitamin D3 (25D) [33]. In this setting, DC expressing the vitamin D receptor (VDR) respond to locally generated 1,25D by promote a tolerogenic phenotype that is associated with enhanced glycolysis, OXPHOS, and TCA metabolism [12]. However, in the presence of elevated levels of 25D, intracrine-generated 1,25D may exit the DC and interact with VDR-expressing T cells within the local immune microenvironment. The effect of 1,25D in this paracrine setting is also tolerogenic and anti-inflammatory [8] but data presented in this study indicate that this is associated with entirely different metabolic programming to that observed for intracrine effect of 1,25D on DC. This flexibility in metabolic regulation by 1,25D in DC and T cells may help to provide a more robust immune response, particularly under conditions of vitamin D repletion.

Heterogenous populations of bulk CD4^+^ T cells are used in this study, including Tregs which have a markedly different metabolism to Th1, Th2, or Th17 cells [34,35]. Future studies may investigate whether 1,25D-mediated metabolic suppression has stronger functional effects on individual subsets, namely Th1 and Th17 cells, which rely heavily on glycolysis [34]. We have only explored the effect of 1,25D on CD4^+^ T-cell monocultures. However, T cell exposure to 1,25D in vivo is dependent on APCs, with APCs synthesizing increased 1,25D in response to T cells [36]. It remains unclear how metabolic effects of 1,25D on both cell types may affect APC/T cell interactions and function. Here, we focussed our investigation on the control of CD4^+^ T cell IFN-γ expression, since this is known to be closely linked to metabolic activity. However, there are likely additional implications of 1,25D-mediated metabolic suppression for T cells. For example, inhibition of glycolysis may impair their survival, motility, and other immune functions, particularly in hypoxic and glucose-deprived tissue environments. Overall, understanding metabolic aspects of 1,25D-mediated immune regulation will aid to define its potential for therapeutic use, and may inform novel strategies in combination with metabolic mediators.

## Supplementary Material

supinfo2

supinfo1

## References

[1] Dankers W, Colin EM, van Hamburg JP, Lubberts E. Vitamin D in autoimmunity: molecular mechanisms and therapeutic potential. Frontiers in Immunology. 2017;7:697. 28163705 10.3389/fimmu.2016.00697 PMC5247472

[2] Bishop E, Ismailova A, Dimeloe SK, Hewison M, White JH. Vitamin D and immune regulation: antibacterial, antiviral, anti-inflammatory. JBMR Plus. 2020;1:e10405. 10.1002/jbm4.10405 PMC7461279 32904944

[3] Unger WWJ, Laban S, Kleijwegt FS, Van Der Slik AR, Roep BO. Induction of Treg by monocyte-derived DC modulated by vitamin D3 or dexamethasone: differential role for PD-L1. Eur J Immunol. 2009;39(11):3147–59. 19688742 10.1002/eji.200839103

[4] Ferreira GB, Vanherwegen A-S, Eelen G, Gutiérrez ACF, Van Lommel L, Marchal K, Vitamin D3 induces tolerance in human dendritic cells by activation of intracellular metabolic pathways. Cell Rep. 2015;10(5):711–25. 25660022 10.1016/j.celrep.2015.01.013

[5] Vanherwegen A-S, Eelen G, Ferreira GB, Ghesquière B, Cook DP, Nikolic T, Vitamin D controls the capacity of human dendritic cells to induce functional regulatory T cells by regulation of glucose metabolism. J Steroid Biochem Mol Biol. 2019;1(187):134–45. 10.1016/j.jsbmb.2018.11.011 30481575

[6] Jeffery LE, Qureshi OS, Gardner D, Hou TZ, Briggs Z, Soskic B, Vitamin D Antagonises the suppressive effect of inflammatory cytokines on CTLA-4 expression and regulatory function. PloS one. 2015;10(7):e0131539. 10.1371/journal.pone.0131539 PMC4489761 26134669

[7] Cantorna MT, Snyder L, Lin YD, Yang L. Vitamin D and 1,25 (OH)2D regulation of T cells. Nutrients. 2015;7:3011–21. 25912039 10.3390/nu7043011 PMC4425186

[8] Jeffery LE, Burke F, Mura M, Zheng Y, Qureshi OS, Hewison M, 1,25-Dihydroxyvitamin D3 and IL-2 combine to inhibit T cell production of inflammatory cytokines and promote development of regulatory T cells expressing CTLA-4 and FoxP3. Journal of Immunology. 2009;183(9):5458–67. 10.4049/jimmunol.0803217 PMC2810518 19843932

[9] Joshi S, Pantalena L-C, Liu XK, Gaffen SL, Liu H, Rohowsky-Kochan C, 1,25-Dihydroxyvitamin D3 ameliorates Th17 autoimmunity via transcriptional modulation of interleukin-17A. Mol Cell Biol. 2011;31(17):3653–69. 21746882 10.1128/MCB.05020-11 PMC3165548

[10] Urry Z, Chambers ES, Xystrakis E, Dimeloe S, Richards DF, Gabryšová L, The role of 1α,25-dihydroxyvitamin D3 and cytokines in the promotion of distinct Foxp3+and IL-10+ CD4+ T cells. Eur J Immunol. 2012;42(10):2697–708. 22903229 10.1002/eji.201242370 PMC3471131

[11] Muñoz Garcia A, Kutmon M, Eijssen L, Hewison M, Evelo CT, Coort SL. Pathway analysis of transcriptomic data shows immunometabolic effects of vitamin D. J Mol Endocrinol. 2018;60(2):95–108. 29233860 10.1530/JME-17-0186 PMC5850959

[12] Garcia AM, Bishop EL, Li D, Jeffery LE, Garten A, Thakker A, Tolerogenic effects of 1,25-dihydroxyvitamin D on dendritic cells involve induction of fatty acid synthesis. J Steroid Biochem Mol Biol. 2021;211:105891. 10.1016/j.jsbmb.2021.105891 PMC8223499 33785437

[13] Riek AE, Oh J, Bernal-Mizrachi C. Vitamin D regulates macro-phage cholesterol metabolism in diabetes. J Steroid Biochem Mol Biol. 2010;121(1–2):430–3. 20338238 10.1016/j.jsbmb.2010.03.018

[14] Riek AE, Oh J, Darwech I, Worthy V, Lin X, Ostlund RE, Vitamin D 3 supplementation decreases a unique circulating monocyte cholesterol pool in patients with type 2 diabetes. J Steroid Biochem Mol Biol. 2018;177:187–92. 28941998 10.1016/j.jsbmb.2017.09.011 PMC5826751

[15] Frauwirth KA, Riley JL, Harris MH, Parry RV, Rathmell JC, Plas DR, The CD28 signaling pathway regulates glucose metabolism. Immunity. 2002;16(6):769–77. 12121659 10.1016/s1074-7613(02)00323-0

[16] Lunt SY, Vander Heiden MG. Aerobic glycolysis: meeting the metabolic requirements of cell proliferation. Annu Rev Cell Develop Biol. 2011;27:441–64. 10.1146/annurev-cellbio-092910-154237 21985671

[17] Chang CH, Curtis JD, Maggi LB, Faubert B, Villarino AV, O’Sullivan D, Posttranscriptional control of T cell effector function by aerobic glycolysis. Cell. 2013;153(6):1239–51. 23746840 10.1016/j.cell.2013.05.016 PMC3804311

[18] Peng M, Yin N, Chhangawala S, Xu K, Leslie CS, Li MO. Aerobic glycolysis promotes T helper 1 cell differentiation through an epigenetic mechanism. Science. 2016;354(6311): 481–4. 27708054 10.1126/science.aaf6284 PMC5539971

[19] Zeitelhofer M, Adzemovic MZ, Gomez-Cabrero D, Bergman P, Hochmeister S, N’diaye M, Functional genomics analysis of Vitamin D effects on CD4+ T cells in vivo in experimental autoimmune encephalomyelitis. Proc Natl Acad Sci U S A. 2017;114(9):E1678–87. 28196884 10.1073/pnas.1615783114 PMC5338504

[20] Berge T, Leikfoss IS, Brorson IS, Bos SD, Page CM, Gustavsen MW, The multiple sclerosis susceptibility genes TAGAP and IL2RA are regulated by vitamin D in CD4+ T cells. Genes Immun. 2016;17:118–27. 26765264 10.1038/gene.2015.61 PMC4783434

[21] Karmali R, Hewison M, Rayment N, Farrow SM, Brennan A, Katz DR, 1,25(OH)2D3 regulates c-myc mRNA levels in tonsillar T lymphocytes. Immunology. 1991;74(4):589–93. 1783418 PMC1384765

[22] Calton EK, Keane KN, Raizel R, Rowlands J, Soares MJ, Newsholme P. Winter to summer change in vitamin D status reduces systemic inflammation and bioenergetic activity of human peripheral blood mononuclear cells. Redox Biol. 2017; 12:814–20. 28441630 10.1016/j.redox.2017.04.009 PMC5406546

[23] Calton EK, Keane KN, Soares MJ, Rowlands J, Newsholme P. Prevailing vitamin D status influences mitochondrial and glycolytic bioenergetics in peripheral blood mononuclear cells obtained from adults. Redox Biol. 2016;10:243–50. 27816874 10.1016/j.redox.2016.10.007 PMC5097975

[24] Dimeloe S, Gubser P, Loeliger J, Frick C, Develioglu L, Fischer M, Tumor-derived TGF-β inhibits mitochondrial respiration to suppress IFN-γ production by human CD4+ T cells. Sci Signal. 2019;12(599):eaav3334. 10.1126/scisignal.aav3334 31530731

[25] Wang R, Dillon CP, Shi LZ, Milasta S, Carter R, Finkelstein D, The transcription factor Myc controls metabolic reprogramming upon T lymphocyte activation. Immunity. 2011;35: 871–82. 22195744 10.1016/j.immuni.2011.09.021 PMC3248798

[26] Marchingo JM, Sinclair LV, Howden AJ, Cantrell DA. Quantitative analysis of how Myc controls T cell proteomes and metabolic pathways during t cell activation. Elife. 2020;9:e53725. 10.7554/eLife.53725 PMC7056270 32022686

[27] Dankers W, Davelaar N, van Hamburg JP, van de Peppel J, Colin EM, Lubberts E. Human memory Th17 cell populations change into anti-inflammatory cells with regulatory capacity upon exposure to active Vitamin D. Front Immunol. 2019;10: 1504. 31379807 10.3389/fimmu.2019.01504 PMC6651215

[28] Chang SH, Chung Y, Dong C. Vitamin D suppresses Th17 cytokine production by inducing C/EBP homologous protein (CHOP) expression. J Biol Chem. 2010;285(50):38751–5. 20974859 10.1074/jbc.C110.185777 PMC2998156

[29] Salehi-Tabar R, Nguyen-Yamamoto L, Tavera-Mendoza LE, Quail T, Dimitrov V, An B-S, Vitamin D receptor as a master regulator of the c-MYC/MXD1 network. Proc Natl Acad Sci U S A. 2012;109(46):18827–32. 23112173 10.1073/pnas.1210037109 PMC3503153

[30] Xu K, Yin N, Peng M, Stamatiades EG, Shyu A, Li P, Glycolysis fuels phosphoinositide 3-kinase signaling to bolster T cell immunity. Science. 2021;371(6527):405–10. 33479154 10.1126/science.abb2683 PMC8380312

[31] Xu K, Yin N, Peng M, Stamatiades EG, Chhangawala S, Shyu A, Glycolytic ATP fuels phosphoinositide 3-kinase signaling to support effector T helper 17 cell responses. Immunity. 2021;54(5):976–87. 33979589 10.1016/j.immuni.2021.04.008 PMC8130647

[32] Cippitelli M, Santoni A. Vitamin D3: a transcriptional modulator of the interferon-γ gene. Eur J Immunol. 1998;28(10):3017–30. 9808170 10.1002/(SICI)1521-4141(199810)28:10<3017::AID-IMMU3017>3.0.CO;2-6

[33] Hewison M, Freeman L, Hughes SV, Evans KN, Bland R, Eliopoulos AG, Differential regulation of vitamin D receptor and its ligand in human monocyte-derived dendritic cells. J Immunol. 2003;170(11):5382–90. 12759412 10.4049/jimmunol.170.11.5382

[34] Michalek RD, Gerriets VA, Jacobs SR, Macintyre AN, MacIver NJ, Mason EF, Cutting edge: distinct glycolytic and lipid oxidative metabolic programs are essential for effector and regulatory CD4+ T cell subsets. J Immunol. 2011;186: 3299–303. 21317389 10.4049/jimmunol.1003613 PMC3198034

[35] Cluxton D, Petrasca A, Moran B, Fletcher JM. Differential regulation of human Treg and Th17 cells by fatty acid synthesis and glycolysis. Front Immunol. 2019;10:115. 30778354 10.3389/fimmu.2019.00115 PMC6369198

[36] Jeffery LE, Wood AM, Qureshi OS, Hou TZ, Gardner D, Briggs Z, Availability of 25-hydroxyvitamin D 3 to APCs controls the balance between regulatory and inflammatory T cell responses. J Immunol. 2012;189(11):5155–64. 23087405 10.4049/jimmunol.1200786 PMC3504609

