# Supplementary material for: 1,25-Dihydroxyvitamin D3 suppresses CD4+ T-cell effector functionality by inhibition of glycolysis
Source: Immunology. Author manuscript; Available in PMC 2023 Jul 1. (PMC9232967; doi:10.1111/imm.13472)
Supplement: supinfo2 [file NIHMS1790452-supplement-supinfo2.docx]

| **Gene target** | **Primer sequence** |
| --- | --- |
| PPARA | FWD: AGAGATTTCGAAATCCATGC |
|  | REV: ACTGGTATTCCGTAAAGCCAAAG |
| PRC | FWD: GAGCAGGTTATCTCTGGAGGA |
|  | REV: GTGAGCAGCGACACTTCATT |
| TFAM | FWD: GAACAACTACCCATATTTAAAGCTCA |
|  | REV: GAATCAGGAAGTTCCCTCCA |
| NRF1 | FWD: TGGCTACTTACACCGAGCATA |
|  | REV: AGAAGGCGAGTCTTCATCAGC |
| CMYC | FWD: AAAGGCCCCCAAGGTAGTTA |
|  | REV: GCACAAGAGTTCCGTAGCTG |
| IFNG | FWD: CTTTAAAGATGACCAGAGCATCCA |
|  | REV: ATCTCGTTTCTTTTTGTTGCTATTGA |
| GLUT1 | FWD: AGGTGATCGAGGAGTTCTAC |
|  | REV: TCAAAGGACTTGCCCAGTTT |
| PHGDH | FWD: GGTCTGCCCTGGAATTGAACT |
|  | REV: CCCTCTGTATGGTGCAGATCC |

**Table S1: Details of primers used for qPCR**
