# Supplementary material for: 1,25-Dihydroxyvitamin D3 suppresses CD4+ T-cell effector functionality by inhibition of glycolysis
Source: Immunology. Author manuscript; Available in PMC 2023 Jul 1. (PMC9232967; doi:10.1111/imm.13472)
Supplement: supinfo1 [file NIHMS1790452-supplement-supinfo1.docx]

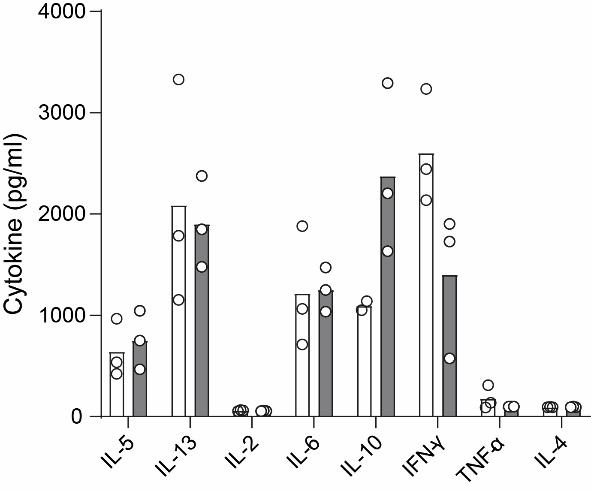


**Supplementary Figure 1: 1,25D inhibits CD4^+^ T cell IFN-γ expression and promotes IL-10 expression at day 5 of culture.**

**
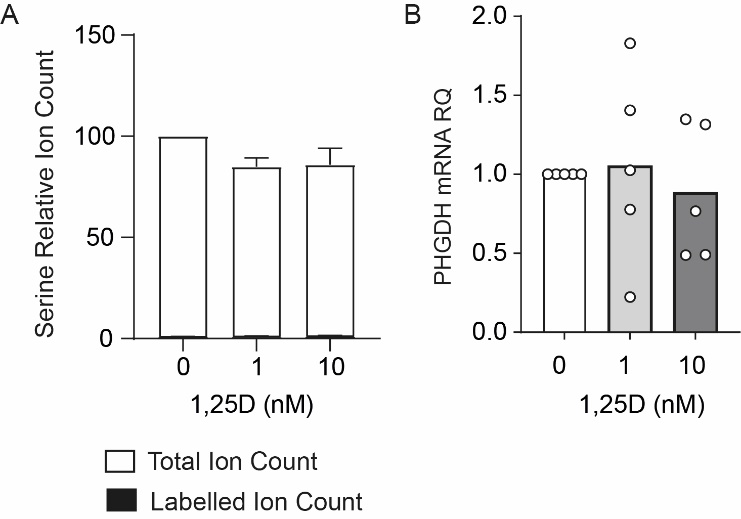
**Human primary CD4^+^ T cells were stimulated in presence of 0, 1 or 10 nM 1,25D as indicated for 5 days and assessed for secretion of the indicated cytokines by LEGENDplex analysis of cell culture supernatants (n = 3 independent donors).

**Supplementary Figure 2: 1,25D inhibits does not affect serine synthesis from glucose in CD4+ T cells**

(A) Human primary CD4^+^ T cells were stimulated in presence of 0, 1 or 10 nM 1,25D as indicated for 48 hours, washed, and then incubated with fully 13C-labelled glucose for 6h and assessed for total and 13-C-labelled ion counts of serine by GC-MS (n = 5 independent donors, data for 1,25D-treated cells are normalised to non-treated controls within each experiment). (B) (CD4^+^ T cells were cultured as in (A) and assessed for mRNA abundance of PHGDH by qPCR. (n = 5 independent donors, data for 1,25D-treated cells are normalised to non-treated controls within each experiment)


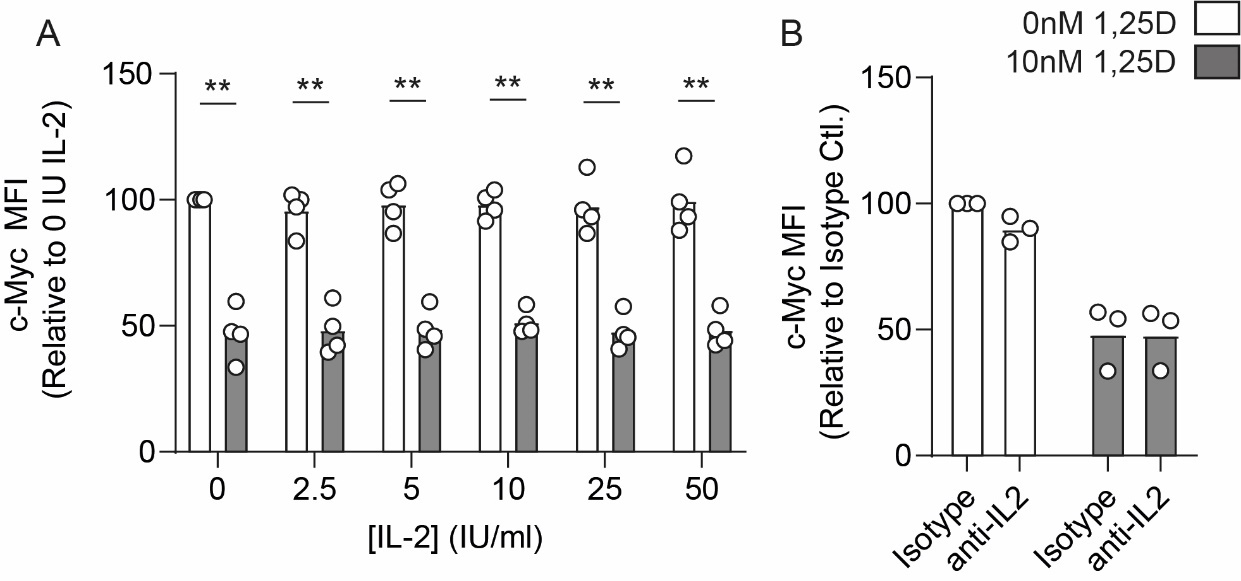


**Supplementary Figure 3: 1,25D inhibits expression of c-Myc independently of effects on IL-2 expression.**

(A-B) Human primary CD4+ T cells were stimulated in presence of 0 or 10 nM 1,25D as indicated, and additionally in presence of a range of concentration of recombinant IL-2, or anti-IL-2 antibody/isotype control as indicated for 48 hours and assessed for C-Myc protein expression by flow cytometry, expressed as mean fluorescence intensity (MFI) (A, n= 4 independent donors, B n = 3 independent donors) **p<0.01.


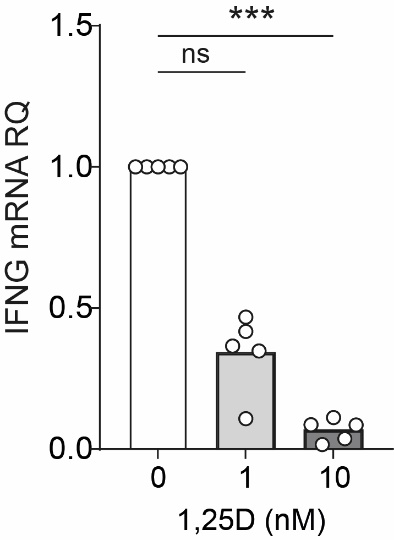


**Supplementary Figure 4: Transcriptional repression of IFNG expression is maximal at 10 nM 1,25D**

(A) Human primary CD4^+^ T cells were stimulated in presence of 0, 1 or 10 nM 1,25D as indicated for 48 hours and assessed for mRNA abundance of IFNG by qPCR. (n = 5 independent donors, data for 1,25D-treated cells are normalised to non-treated controls within each experiment)
